# Supplementary figures and images for: Predictive Roles of Basal Metabolic Rate and Muscle Mass in Lung Function among Patients with Obese Asthma: A Prospective Cohort Study
Source: Nutrients. 2024 Jun 8;16(12):1809. doi: 10.3390/nu16121809 (PMC11206345; doi:10.3390/nu16121809)

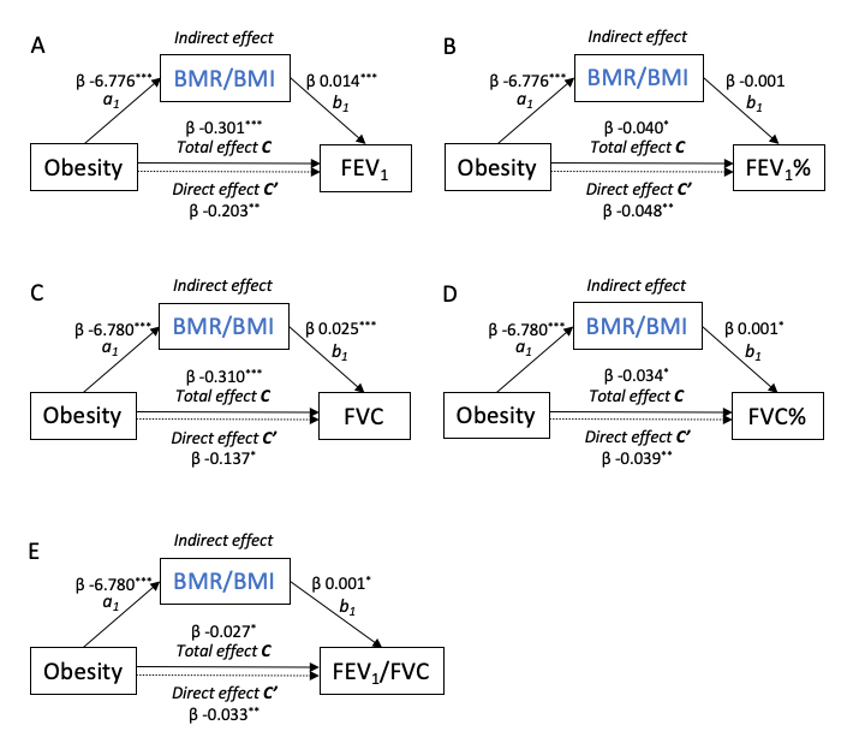

Supplement: Supplementary file 1 [file nutrients-16-01809-s001.zip › Figure S1.png]

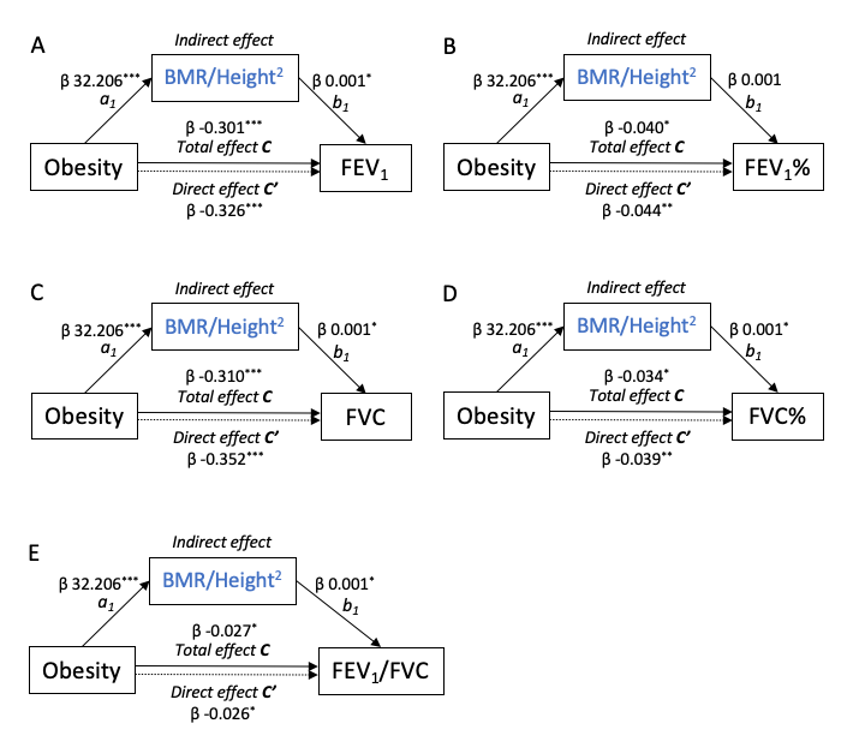

Supplement: Supplementary file 1 [file nutrients-16-01809-s001.zip › Figure S2.png]

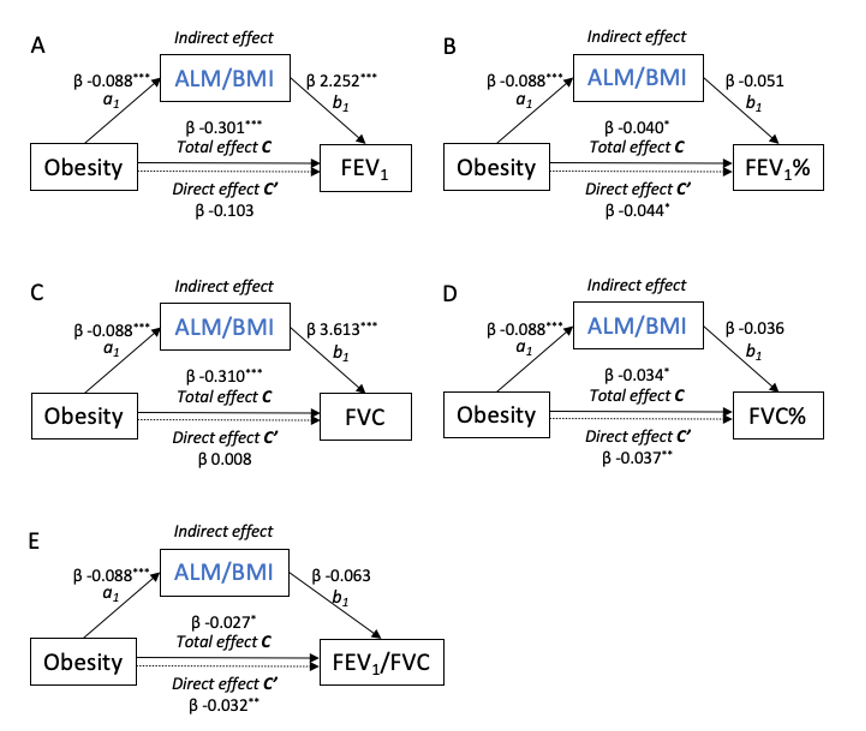

Supplement: Supplementary file 1 [file nutrients-16-01809-s001.zip › Figure S3.png]

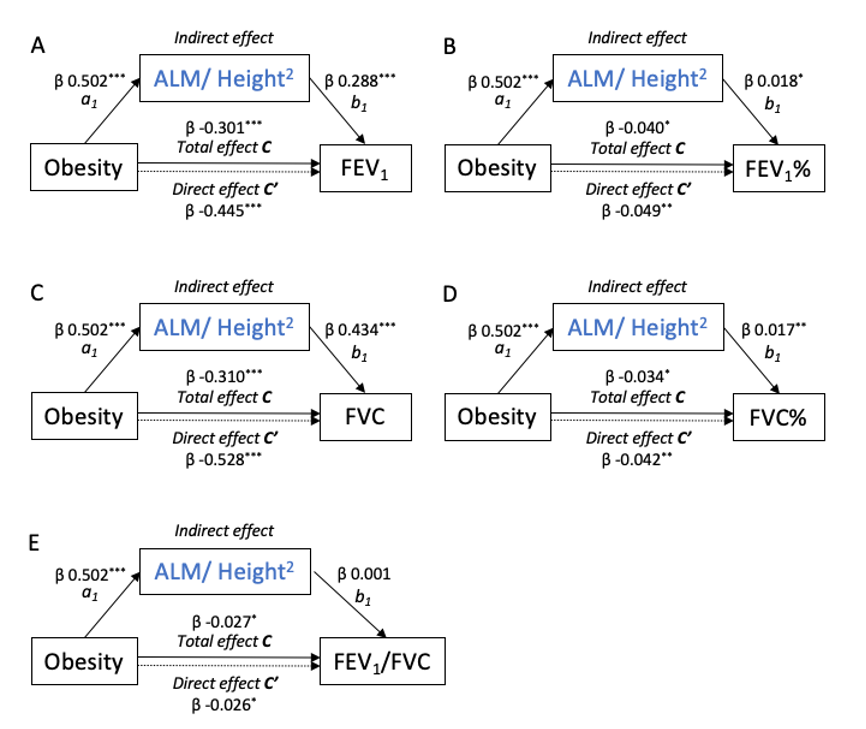

Supplement: Supplementary file 1 [file nutrients-16-01809-s001.zip › Figure S4.png]

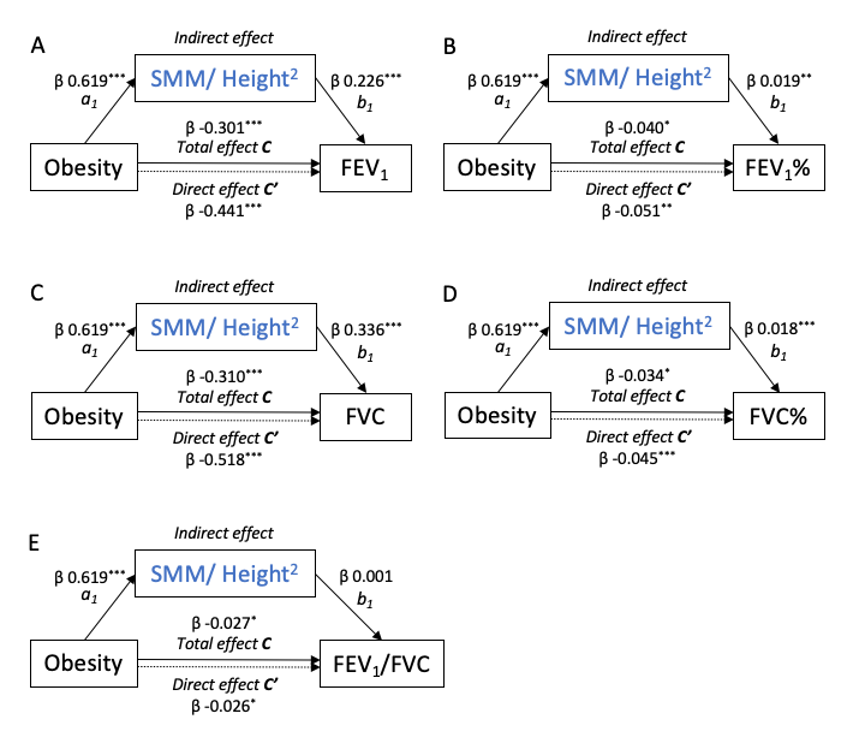

Supplement: Supplementary file 1 [file nutrients-16-01809-s001.zip › Figure S5.png]

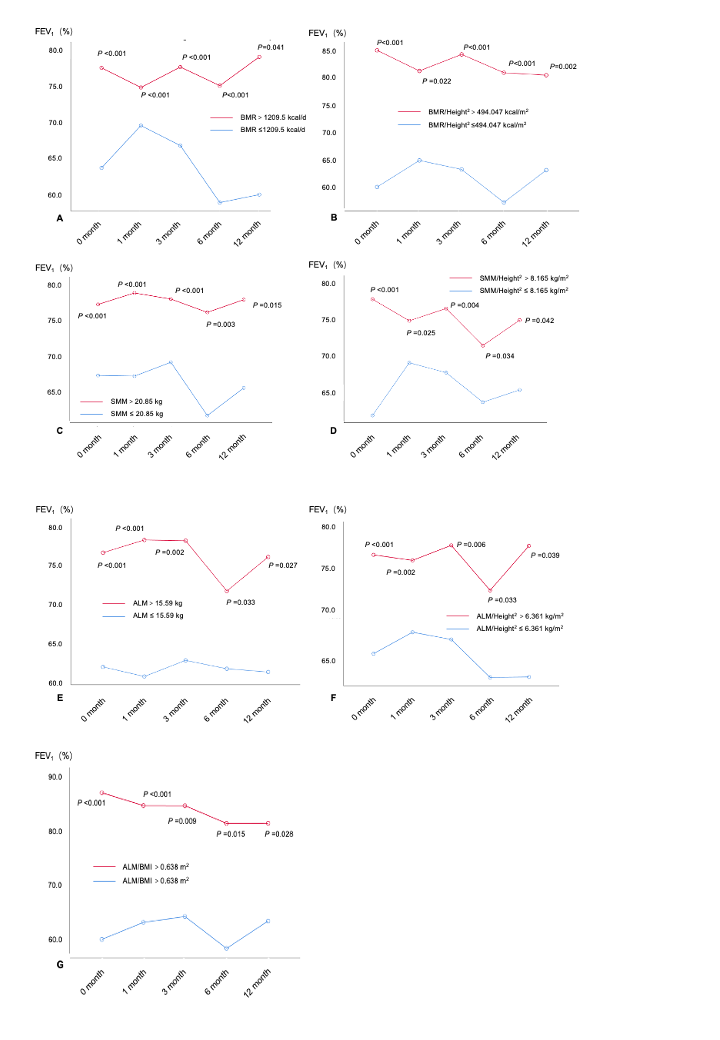

Supplement: Supplementary file 1 [file nutrients-16-01809-s001.zip › Figure S6.png]

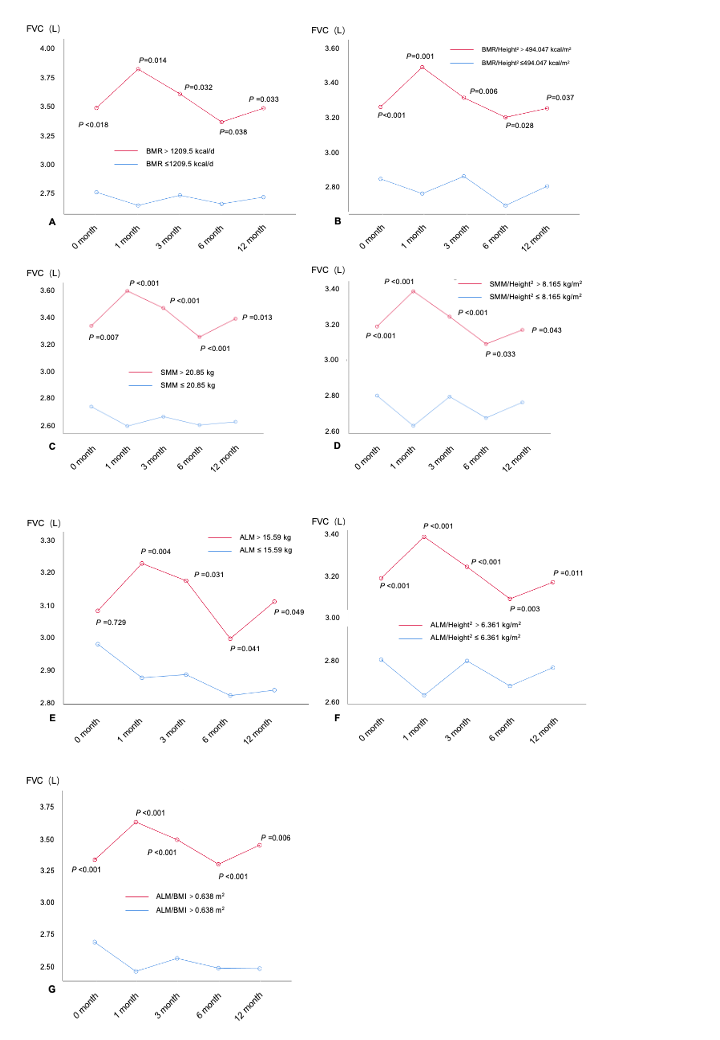

Supplement: Supplementary file 1 [file nutrients-16-01809-s001.zip › Figure S7.png]

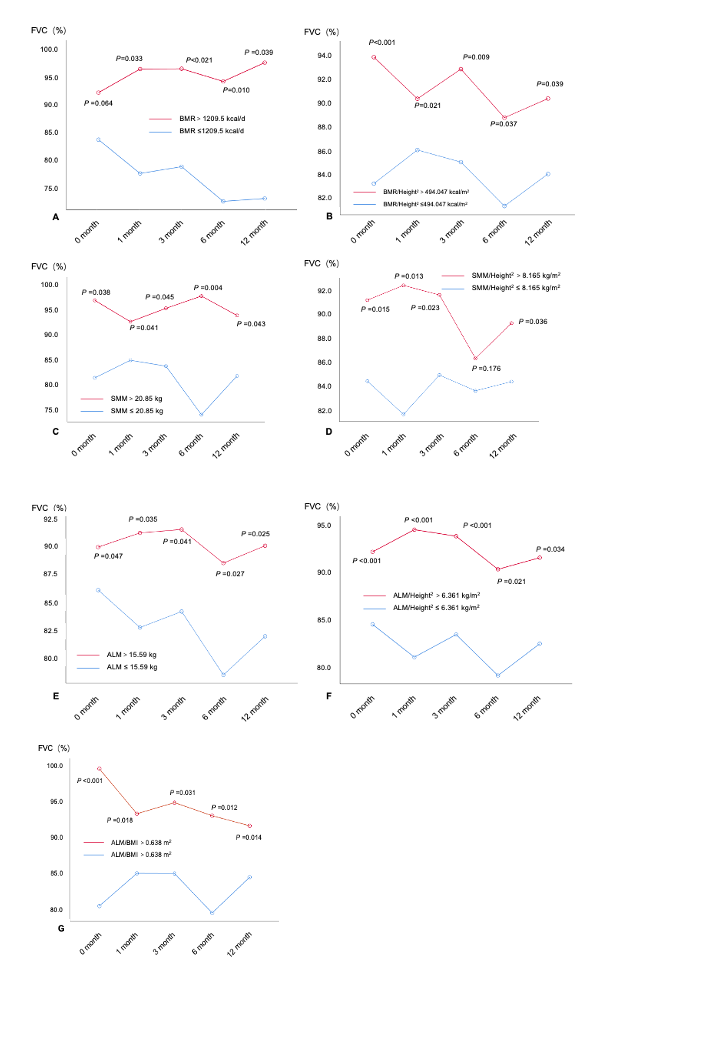

Supplement: Supplementary file 1 [file nutrients-16-01809-s001.zip › Figure S8.png]
